# Synthesis of Cellulose Nanocrystals/HKUST-1 Composites and Their Applications: Crystal Violet Removal and Doxorubicin Loading

**DOI:** 10.3390/polym14224991

**Published:** 2022-11-18

**Authors:** Christian J. Wijaya, Felycia E. Soetaredjo, Suryadi Ismadji, Setiyo Gunawan

**Affiliations:** 1 Department of Chemical Engineering, Faculty of Industrial Technology and Systems Engineering, Institut Teknologi Sepuluh Nopember, Keputih Sukolilo, Surabaya 60111, Indonesia; 2 Department of Chemical Engineering, Widya Mandala Surabaya Catholic University, Kalijudan 37, Surabaya 60114, Indonesia; 3 Collaborative Research Center for Zero Waste and Sustainability, Widya Mandala Surabaya Catholic University, Kalijudan 37, Surabaya 60114, Indonesia; 4 Department of Chemical Engineering, National Taiwan University of Science and Technology, 43 Keelung Road, Section 4, Taipei 10607, Taiwan

**Keywords:** cellulose nanocrystals, composite, drug loading, dye removal, metal–organic frameworks

## Abstract

This study developed a novel composite material containing cellulose nanocrystals (CNCs) and HKUST-1. Here, the addition of CNCs was used to enhance the characteristics of HKUST-1 in terms of surface area, adsorption ability, and functional groups. Here, the fabrication of CNCs@HKUST-1 composites was carried out by adding CNCs into the fabrication process of HKUST-1. The addition of CNCs provides additional functional groups on the surface of composite material which can be used to attach other organic compounds, such as in waste management and drug delivery systems. Here, CNCs@HKUST-1 composites were tested as a material for crystal violet (CV) removal and doxorubicin (DOX) loading. The removal capacity of CNCs@HKUST-1 composite towards CV molecules reached 1182.25 ± 27.74 mg/g, while the loading capacity for DOX drugs was around 1514.94 ± 11.67 mg/g. Both applications showed that CNCs@HKUST-1 composite had higher adsorption capacity and ability compared to its precursor materials, i.e., CNCs and HKUST-1.

## 1. Introduction

Cellulose nanocrystals (CNCs) are porous crystalline materials that are biodegradable, biocompatible, nontoxic, and appliable in various applications including composites, adsorbents, waste management systems, and drug delivery systems [1,2]. In addition, CNCs are materials that can be used in the functionalization of other materials so that they are suitable as a composite composition. In a composite framework, CNCs can provide larger surface area, higher biodegradability, and additional reactive functional groups. Here, CNCs can be integrated with metal–organic frameworks (MOFs) to fabricate an advanced composite which can be further used in various applications.

MOFs are advanced materials formed from metal and organic ligands that form a two−dimensional and three−dimensional structural framework. MOFs are porous crystalline materials that have large surface area, good thermal stability, adaptable structure, and various functions [3,4]. In addition, MOFs are easily modified with other materials to suit specific application requirements [5]. However, the applications of MOFs have their own challenges, namely, (1) tendency to have a low electrical conductivity so that it does not have a positive or negative charge [6], (2) tendency to be more brittle so it is difficult to maintain its macroscopic shape and hierarchical porosity [4], (3) tendency to agglomerate easily, (4) tendency to have low stability in water, and (5) tendency to be difficult to reuse [3]. Here, composite fabrication by combining CNCs with MOFs is an attempt to overcome the challenges of MOFs applications. The integration between CNCs and MOFs can be formed by an in situ process wherein CNCs are added during the fabrication process of MOFs and are trapped in the framework of MOFs. This in situ process is projected to build novel CNCs-integrated MOFs (CNCs@MOFs) with better characteristics than their precursors.

CNCs@MOFs can be used in many applications, such as wastewater treatment and drug delivery systems. Both represent applications in the environmental and medical fields. The development of these two fields continues to be carried out in line with the acceleration of industrial progress and the condition of the world health system. In these two applications, CNCs@MOFs take on the role of absorbent material for both waste and medicine. Naturally, the mechanism and characteristics of the application of CNCs@MOFs in the two applications will be slightly different based on various aspects that affect these applications.

In wastewater treatment, CNCs@MOFs are used as adsorbents to trap waste molecules, such as dyes, chemicals, and oils, which can come from industries, households, and nature. In this application, this material must have a high adsorption ability, a sturdy and strong structure, and be easy to reuse. The textile industry is one example of an industry that produces colored liquid waste containing several dye molecules used in its production. This needs to be addressed to prevent environmental problems, such as in rivers, reservoirs, and oceans. This material is expected to be used to absorb dye molecules in wastewater so that the wastewater can be discharged into the environment by meeting the specified quality standards.

In another application, CNCs@MOFs can also be used in drug delivery systems where drugs can be delivered into the human body by being loaded on this drug carrier material. The characteristics of the drug carrier must be adapted to the mode and mechanism of drug administration, such as intravenous (IV), intramuscular (IM), intranasal (IN), intradermal (ID), or oral. The administration of chemotherapy drugs is a current concern to be developed to be more effective, safe, and easy for cancer patients. This material can be used to load and deliver chemotherapy drugs into the human body through an oral mechanism to reduce the side effects of chemotherapy drugs, such as nausea, vomiting, hair loss, and blackening nails.

In this study, CNCs were used to modify HKUST-1, one type of MOF which has great characteristics for adsorption applications. Here, the room−temperature fabrication process was carried out in situ to obtain the well−integrated frameworks of CNCs and HKUST-1. The use of room temperature is an effort to save energy compared to the use of high temperature and pressure, as has been performed in previous studies. Further, the composite product was referred to as the CNCs@HKUST-1 composite and was then used for preliminary studies of crystal violet (CV) removal and doxorubicin (DOX) loading. Two applications were tested here to prove the wide applicability of this composite material. Furthermore, this study is expected to underlie technological developments in the fields of wastewater management and medical treatment.

## 2. Materials and Methods

### 2.1. Materials

All materials were purchased from Sigma−Aldrich (Singapore): Whatman paper (grade 40), sulfuric acid (H_2_SO_4_, 98%), copper (II) nitrate hemi(pentahydrate) (Cu(NO_3_)_2_·2.5H_2_O, 98%), benzene-1,3,5-tricarboxylic acid (H_3_BTC, 95%), ethanol (C_2_H_5_OH, 99.5%), and crystal violet (CV) dye. All chemicals were in analytical grade and used without further purification.

### 2.2. Isolation of CNCs

This step followed the procedure optimized by [7,8] without any pretreatment because of the use of Whatman paper, which contains ≥98% cellulose. CNCs were isolated with an acid hydrolysis process using a 55 wt.% sulfuric acid solution at 39 °C. Crushed Whatman paper (5 g) was soaked and stirred in 100 mL of the sulfuric acid solution for 60 min until the solution became a clear yellowish solution. Then, 10−fold cold distilled water was added to stop the hydrolysis process while a cloudy suspension was formed. After that, the solids were separated from the suspension using a centrifuge (3114× *g*) at 6000 rpm for 10 min. The solids were washed with distilled water until the pH was neutral. Eventually, the final suspension was sonicated for 20 min to produce homogenous CNCs and then dried using a freeze dryer at 0.08 mbar and −42 °C.

### 2.3. Preparation of HKUST-1

Here, HKUST-1 was produced with a room−temperature coordination modulation method established by [9]. Two precursor solutions were prepared separately, i.e., (A) 40 mL of 0.05 M Cu(NO_3_)_2_·2.5H_2_O in 5% *v*/*v* acetic acid solution and (B) 40 mL of 0.0235 M H_3_BTC in 1:1 ethanol–water solution. Then, solution B was added dropwise into solution A while a cloudy turquoise suspension was formed. This suspension was constantly stirred for 27.2 h at room temperature. After that, the produced solids were separated from the suspension using a centrifuge (3114× *g*) at 6000 rpm for 5 min. Next, the solids were washed twice with ethanol and dried using an oven at 70 °C.

### 2.4. Fabrication of CNCs@HKUST-1 Composites

CNCs@HKUST-1 composites were fabricated in situ where CNCs were added during HKUST-1 preparation. The addition of CNCs was varied by 2.5, 5, 7.5, and 10 wt.% of the metal precursor in the HKUST-1 preparation. This varied parameter was proposed to investigate the best composition of CNCs and HKUST-1 in the composite framework. Here, CNCs were mixed into solution A before the dropwise addition of solution B. The next steps followed the HKUST-1 preparation in Section 2.3. These composite products were coded as CNCs@HKUST-1(2.5), CNCs@HKUST-1(5), CNCs@HKUST-1(7.5), and CNCs@HKUST-1(10) according to the percentage addition of CNCs. Further, all CNCs, HKUST-1, and CNCs@HKUST-1 composites were characterized and tested for two different applications, i.e., CV removal and DOX loading. Here, the best condition for the integration of CNCs and HKUST-1 was determined by the adsorption capacity toward CV removal and DOX loading.

### 2.5. Characterizations

All materials, i.e., CNCs, HKUST-1, and CNCs@HKUST-1 composites, were characterized to know their characteristics using scanning electron microscopy (SEM), X-ray diffraction (XRD), and Fourier−transform infrared spectroscopy (FTIR). SEM analysis was performed with a JSM−6390 field emission SEM (Jeol Ltd., Tokyo, Japan). This instrument was operated at 10 kV of accelerating voltage and 7.5 mm of working distance to obtain the best morphological images of all materials. XRD analysis was carried out to investigate the crystal pattern of all materials with a PANalytical X’Pert Pro X-ray diffractometer (Philips−FEI, Eindhoven, The Netherlands). It was conducted using Cu Kα_1_ radiation at 40 kV of voltage, 30 mA of current, and 0.02°/C of step size. FTIR analysis was performed to explore the functional groups of all materials which were conducted with an FTIR Shimadzu 8400S (Shimadzu Scientific Instruments Inc., Seattle, WA, USA) under the KBr pelleting method.

### 2.6. Applications

#### 2.6.1. Crystal Violet (CV) Removal

CV removal was carried out using CNCs, HKUST-1, and CNCs@HKUST-1 composites as the adsorbent by the adsorption method. Here, batch adsorption was performed for each material using an aqueous CV solution in three replications. The adsorbent (10 mg) was mixed into 10 mL of 1264.61 mg/L aqueous CV solution for 24 h at room temperature. Next, the spent adsorbent was separated from the supernatant whose concentration was further measured using a UV/Vis Spectrophotometer Genesys 150 (Thermo Fisher Scientific Inc., Waltham, MA, USA). Then, the removal capacity ($q_{removal}$) was calculated using the following equation (Equation (1)):

(1) $$q_{removal}=\frac{\left(C_{i,CV}-C_{f,CV}\right)V_{CV}}{m}$$

where $C_{i,CV}$ and $C_{f,CV}$ are the initial and final concentration of aqueous CV solution, $V_{CV}$ is the volume of aqueous CV solution, and m is the mass of adsorbent.

#### 2.6.2. Doxorubicin (DOX) Loading

A similar procedure of adsorption was also used for DOX loading in three replications, where 152.90 mg/L aqueous DOX solution was used here. The loading capacity ($q_{loading}$) was calculated using the following equation (Equation (2)):

(2) $$q_{loading}=\frac{\left(C_{i,DOX}-C_{f,DOX}\right)V_{DOX}}{m}$$

where $C_{i,DOX}$ and $C_{f,DOX}$ are the initial and final concentration of aqueous DOX solution, $V_{DOX}$ is the volume of aqueous DOX solution, and m is the mass of adsorbent.

## 3. Results and Discussion

### 3.1. Characteristics of CNCs@HKUST-1 Composites

**Figure 1:**
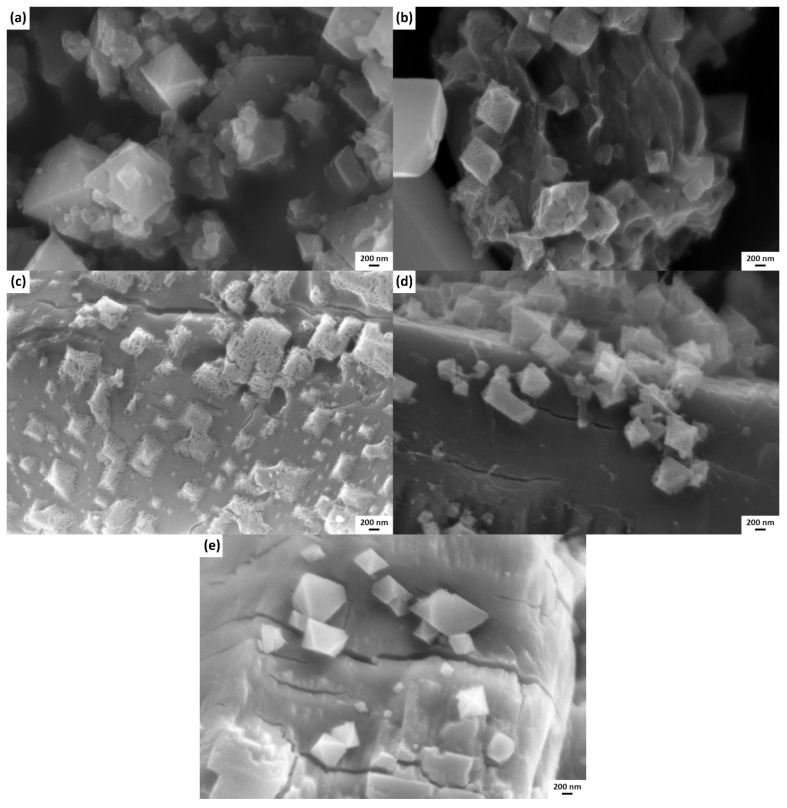
SEM images of (**a**) HKUST-1 and CNCs@HKUST-1 composites with the addition of CNCs of (**b**) 2.5%, (**c**) 5%, (**d**) 7.5%, and (**e**) 10%.

In this study, CNCs@HKUST-1 composites were successfully fabricated by combining CNCs and HKUST-1. Figure 1 shows the morphologies of HKUST-1 and CNCs@HKUST-1 composites with various percentages of CNCs addition. Here, the size of HKUST-1 frameworks in the composite structure was smaller than the single HKUST-1. The presence of CNCs in the HKUST-1 fabrication system causes a decrease in the size of the HKUST-1. Here, CNCs reduce the randomness of the synthesis system so that the collision mechanism of Cu and BTC ions is reduced and the formation of HKUST-1 becomes slower, resulting in a smaller size. In addition, CNCs are also organic compounds that can act as ligands to build a framework together with Cu ions. This provides a benefit to composite material fabrication where CNCs and HKUST-1 can form a well−integrated structure, as shown in Figure 1c. The composite morphology of CNCs@HKUST-1 shows that HKUST-1 was spread over the CNCs framework which had a larger size than HKUST-1. This phenomenon is in accordance with previous research that developed a composite constructed by HKUST-1 and carboxymethyl cellulose (CMC) [10]. Here, the CNCs@HKUST-1(5) composite showed the most uniform and well−integrated morphology. The addition of fewer CNCs makes it difficult to form a CNCs framework that can cover and be integrated with HKUST-1. However, the addition of more CNCs led to the formation of a more solid CNCs frameworks and made it difficult to connect to the HKUST-1.

**Figure 2:**
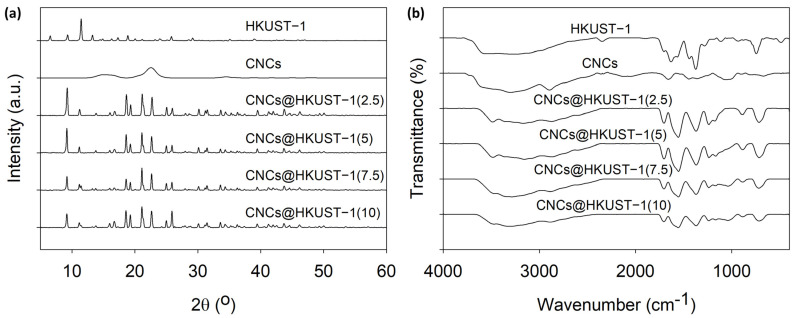
(**a**) XRD diffractogram and (**b**) FTIR spectra of CNCs, HKUST-1, and CNCs@HKUST-1 composites.

In Figure 2a, the XRD diffractogram shows the crystal pattern of CNCs, HKUST-1, and CNCs@HKUST-1 composites. The diffraction patterns in the CNCs@HKUST-1 composite diffractogram are a combination of the diffraction patterns of CNCs and HKUST-1 as precursor materials. This pattern also supports the results of the SEM analysis which shows that the presence of CNCs has a significant effect on the morphology and crystallinity of the CNCs@HKUST-1 composites. Here, the peaks presented above 2θ of 15° indicate the presence of CNCs in the frameworks. In addition, there are two main peaks, i.e., (220) and (222) planes indicated at 2θ around 9.0° and 11.0°, which indicate the diffraction difference between single HKUST-1 and CNCs@HKUST-1 composites. Here, a single HKUST-1 had the (222) plane as the highest peak intensity compared to others, but the highest one was the (220) plane on the diffractograms of CNCs@HKUST-1 composites. It indicates the occurrence of CNCs interactions with Cu ions which forms a more unique and integrated framework of CNCs and HKUST-1 in the composites. On the other side, Figure 2b shows the FTIR spectra of CNCs, HKUST-1, and CNCs@HKUST-1 composites. These spectra prove that all materials had functional groups which came from the precursor material. The fingerprint regions of CNCs@HKUST-1 composites indicate the same functional groups of CNCs and HKUST-1.

### 3.2. Applications of CNCs@HKUST-1 Composites

#### 3.2.1. Crystal Violet (CV) Removal

**Figure 3:**
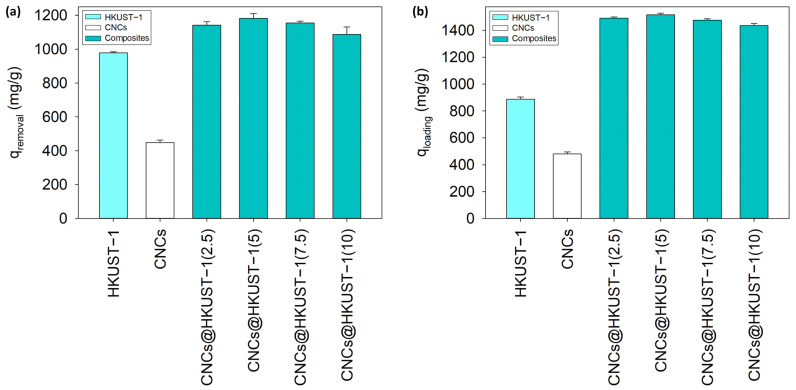
The adsorption capacity of CNCs, HKUST-1, and CNCs@HKUST-1 composites in the applications of (**a**) CV removal and (**b**) DOX loading.

Figure 3a shows the adsorption capacity of CNCs, HKUST-1, and CNCs@HKUST-1 composites at the CNCs addition of 2.5, 5, 7.5, and 10%. In single materials, CNCs have a CV adsorption capacity of up to 448.33 ± 14.55 mg/g which is lower than the adsorption capacity of HKUST-1 (977.99 ± 6.51 mg/g). In the CNCs@HKUST-1 composites, the CNCs addition of 2.5, 5, 7.5, and 10% into the composite frameworks caused an increase in the adsorption capacity of CV up to 16.69, 20.89, 18.08, and 11.15% compared to HKUST-1. Here, it proves that the well−integrated morphology of CNCs@HKUST-1(5) gave the higher ability of CV removal. CNCs@HKUST-1(5) had the ability to adsorb CV compound up to 1182.25 ± 27.74 mg/g, indicating the CNCs addition of 5% as the optimum condition to provide the best CNCs@HKUST-1 composite. As shown in Table 1, the adsorption capacity of CNCs@HKUST-1 composites were higher than other composites consisting of cellulose−based materials and MOFs. Although adsorption is affected by various parameters, these results demonstrate the great potential of utilizing CNCs@HKUST-1 composites in wastewater treatment.

#### 3.2.2. Doxorubicin (DOX) Loading

Here, Figure 3b indicates a similar result of DOX loading with the CV removal where CNCs@HKUST-1(5) performed the highest loading capacity of DOX (1514.94 ± 11.67 mg/g). The capacity increased up to 68.10, 70.79, 66.44, and 61.82% for the CNCs addition of 2.5, 5, 7.5, and 10%, respectively. The increase was almost four times higher than in the application of CV removal. It can happen because DOX has higher amounts of hydroxyl, carbonyl, and amine groups than CV. These functional groups gave better and stronger interactions with the functional groups of CNCs so that the CNCs addition provides the enhancement of DOX loading. Here, Table 1 shows the comparison of various composites that have been used in drug delivery systems. The integration of CNCs and HKUST-1 provided higher drug loading capacity compared to other materials from previous studies.

## 4. Conclusions

Here, this study successfully developed a new composite constructed from CNCs and HKUST-1. The CNCs@HKUST-1 composite has better characteristics compared to its precursor materials, which are suitable for adsorption applications. This composite provides a well−integrated morphology between CNCs and HKUST-1 in one framework. The addition of CNCs enhances the adsorption ability of HKUST-1 in the form of a CNCs@HKUST-1 composite. CNCs provide additional functional groups onto the composite to attach other organic compounds, such as dyes or drugs. In the application of CV removal, CNCs@HKUST-1 composite had an adsorption capacity of 1182.25 ± 27.74 mg/g, which was 20.89% higher than HKUST-1. On another application, this composite provides an outstanding performance for loading DOX with the capacity of 1514.94 ± 11.67 mg/g which was also higher, up to 70.79%, than HKUST-1.

## Figures and Tables

**Table 1:** Comparison of the utilization of integrated cellulose−based materials in MOFs for wastewater treatment and drug delivery systems.

Materials	Applications	Adsorption Capacity	References
In wastewater treatment:			
MCNC@Zn-BTC	Pb(II) removal	558.66 mg/g	[11]
HKUST-1/Fe_3_O_4_/CMF	Methylene blue removal	102 mg/g	[12]
CMC/ZIF-8	Methylene blue removal	13.06 mg/g	[13]
CNCs/ZIF-8	Malachite green removal	1060.2 mg/g	[14]
CNCs@HKUST-1	Crystal violet removal	1182.25 ± 27.74 mg/g	This study
In drug delivery systems:			
ZIF-8/cellulose/Fe_3_O_4_	Glucose oxidase (enzyme) loading	94.26 mg/g	[15]
ZIF-8@TOCNF	Curcumin loading	41 mg/g	[16]
CMC/MOF-5/GO	Doxorubicin loading	64 mg/g	[17]
ZIF-67@CNF	Diclofenac loading	121.20 mg/g	[18]
HKUST-1@CNF		90 mg/g	
CNCs@HKUST-1	Doxorubicin loading	1514.94 ± 11.67 mg/g	This study

Note: MCNC—magnetic cellulose nanocrystals; BTC—benzene−1,3,5−tricarboxylic acid; CMF—cellulose microfibrils; CMC—carboxymethyl cellulose; ZIF-8—zeolitic imidazolate frameworks−8; TOCNF—TEMPO−mediated oxidized cellulose nanofibers; GO—graphene oxide; CNF—cellulose nanofibers.

## Data Availability

Not applicable.
